# Resting hyperinflation and emphysema on the clinical course of COPD

**DOI:** 10.1038/s41598-019-40411-1

**Published:** 2019-03-06

**Authors:** Yeon Wook Kim, Chang-Hoon Lee, Hun-Gyu Hwang, Yu-Il Kim, Deog Kyeom Kim, Yeon-Mok Oh, Sang Haak Lee, Ki Uk Kim, Sang-Do Lee

**Affiliations:** 1 Division of Pulmonary and Critical Care Medicine, Department of Internal Medicine, Seoul National University Hospital, Seoul, Republic of Korea; 2 Department of Medicine, Soonchunhyang University Gumi’s Hospital, North Kyungsang Province, Republic of Korea; 3 Department of Internal Medicine, Chonnam National University Hospital, Gwangju, Republic of Korea; 4 Division of Pulmonary and Critical Care Medicine, Department of Internal Medicine, Seoul National University College of Medicine, Seoul Metropolitan Government-Seoul National University Boramae Medical Center, Seoul, Republic of Korea; 5 Department of Pulmonary and Critical Care Medicine, Asan Medical Center, University of Ulsan College of Medicine, Seoul, Republic of Korea; 6 Department of Internal Medicine, The Catholic University of Korea, St. Paul’s Hospital, Seoul, Republic of Korea; 7 Department of Internal Medicine, Pusan National University School of Medicine, Busan, Republic of Korea

## Abstract

The aim of this study is to clarify whether the combined evaluation of resting hyperinflation and emphysema confers any additional advantages in terms of predicting clinical outcomes in chronic obstructive pulmonary disease (COPD) patients. We included COPD patients from the Korean Obstructive Lung Disease (KOLD) cohort. Patients with a residual volume/total lung capacity (RV/TLC) over the upper limit of normal were defined as having resting hyperinflation, and those with an emphysema index >10% were defined as having emphysema. We investigated the impacts of resting hyperinflation and emphysema on exacerbations and mortality. A total of 310 COPD patients were analyzed over a mean of 61.1 months. After adjustment for covariates, resting hyperinflation was an independent predictor of earlier exacerbation (HR = 1.66, CI = 1.24–2.22), more frequent exacerbation (IRR = 1.35, CI = 1.01–1.81), and higher mortality (HR = 2.45, CI = 1.16–5.17) risk. Emphysema was also significantly associated with earlier exacerbation (HR = 1.64, CI = 1.15–2.35), and higher mortality (HR = 3.13, CI = 1.06–9.27) risk. Participants with both resting hyperinflation and emphysema had an additively higher risk of earlier exacerbations (HR = 1.71, 95% CI = 1.26–2.33) and mortality (HR = 3.75, 95% CI = 1.81–7.73) compared with those in other groups. In conclusion, resting hyperinflation and emphysema had additional worse impacts on exacerbations and mortality in COPD patients.

## Introduction

Chronic obstructive pulmonary disease (COPD), which is characterized by persistent and progressive airflow limitation, is a major cause of morbidity and mortality worldwide^1,2^. Furthermore, the disease is associated with increasing socioeconomic burdens^3^. The main pathophysiology of the disease involves inflammatory responses in the small airways and parenchymal destruction of the lung. However, the contribution of each of these individual factors varies from person to person, and as a result, COPD has various phenotypes^4,5^. The current diagnosis and classification of COPD is based mainly on the degree of expiratory flow limitation, which is represented by the forced expiratory volume in one second (FEV1). However, studies have shown that FEV1 alone cannot explain the inherent complexity of the various COPD phenotypes^6^.

In addition to expiratory airflow limitation, resting hyperinflation is another major manifestation of COPD caused by injury of the small airways and parenchymal destruction, which is known to be closely related to symptoms and exercise performance^7–9^. Indeed, the residual volume/total lung capacity ratio (RV/TLC), which is one of the most commonly used index reflecting the degree of resting hyperinflation with equations for reference values available^10,11^, is a potential prognostic marker associated with mortality in COPD^12–14^. However, small airway dysfunction occurs as a result of normal aging; such dysfunction can also lead to hyperinflation. For this reason, evaluation of the RV/TLC alone can lead to misrepresentations of the true status of COPD^15^. In addition, data remain scarce regarding the relationship between RV/TLC and outcomes other than mortality; in particular, few well-designed prospective studies have addressed this issue.

Another important pathological manifestation of COPD—emphysema—reflects the parenchymal destruction of the lung^16^. The presence and degree of emphysema can be quantified using computed tomography (CT), and the condition is also known to be associated with the various outcomes of COPD^17–19^. However, a significant proportion of patients with emphysema—even those with severe emphysema—show preserved lung function and exercise capacity, with only mild symptoms. Thus, the evaluation of emphysema alone as a prognostic marker also has shortcomings^5,20,21^.

Resting hyperinflation and emphysema are closely related, and they represent the “emphysema-hyperinflated” phenotype of COPD. However, most previous studies have evaluated the relationship of only one of these features with disease outcome^22^. Accordingly, we hypothesized that the evaluation of resting hyperinflation and emphysema in combination would provide additional information regarding the relationship of both features with the clinical course of COPD.

Therefore, we carried out a multicenter, prospective, longitudinal study involving detailed assessment of patients with COPD. The participants were observed for up to 10 years under appropriate treatment^23^. The aim of this study was to assess the associations of resting hyperinflation and emphysema with the clinical course of COPD. Hyperinflation was evaluated using RV/TLC, while emphysema was determined using CT. In addition, this study aimed to explore whether the evaluation of these two features in combination confers additional information along with phenotypical classification for the prediction of exacerbations and mortality in COPD patients.

## Methods

### Study design and participants

The KOLD study prospectively recruited patients with obstructive lung disease from the pulmonary clinics of 14 referral hospitals in South Korea from June 2005 to October 2012. The details of this cohort have been reported previously^23^. The present study included patients from the cohort who (1) were aged ≥40 years; (2) had a post-bronchodilator FEV1/forced vital capacity (FVC) below the lower limits of normal (LLN) according to the prediction equation suggested by the Global Lung Initiative (GLI)^24^; (3) were current or ex-smokers, with a smoking history of more than 10 pack-years; and (4) had the results of both chest CT and body plethysmography available at baseline. Patients with an RV/TLC over the upper limits of normal (ULN) as calculated by prediction equations adjusting for sex and age bias were defined as having resting hyperinflation^10^, and those with an emphysema index (volume fraction of the lung ≤ −950 Hounsfield Units [HU]) >10% were defined as having emphysema^17^. To evaluate whether combined evaluation of the two features would inform better prediction of the clinical outcomes of COPD, subjects were classified into four groups: Group 1 had an RV/TLC ≤ ULN and an emphysema index ≤ 10%; Group 2 had an RV/TLC ≤ ULN and an emphysema index >10%; Group 3 had an RV/TLC > ULN and an emphysema index ≤ 10%; and Group 4 had an RV/TLC > ULN and an emphysema index >10%. First, we independently evaluated the association between each feature—hyperinflation and emphysema—as well as emphysema index and RV/TLC as a continuous variable, and the clinical course of COPD. We then performed multiple comparisons of long-term outcomes among the groups.

The design of this study was approved by the ethics committee of the Seoul National University Hospital Institutional Review Board (IRB no. 1611–013–804). The study was conducted in accordance with the Declaration of Helsinki. Written, informed consent was obtained from all included subjects.

### Procedures and measurements

Baseline clinical data from all participants, including demographic information and smoking status, were collected. To establish symptom scores, St. George’s respiratory questionnaire (SGRQ) and the modified Medical Research Council (mMRC) dyspnea-scale were used. Chronic bronchitis was defined as the presence of phlegm for periods of three months or more for at least two years. Each patient’s history of acute exacerbations in the year prior enrollment was also collected. In addition to regular follow-ups at three-month intervals, reports were taken when patients experienced exacerbations or mortality. Participants visited the clinics every 3 months for assessments of the experience of acute exacerbations. If a participant did not visit the attending clinic, research coordinators called him or her to obtain information. The definition of acute exacerbation used in this study was any event that required an unplanned visit to a clinic or emergency room with or without admission due to acute aggravation of respiratory symptoms. Acute exacerbations were assessed based on both self-reports to questionnaires and the medical records including prescription papers. When loss-to-follow-up occurred, the investigators made reasonable efforts to contact the participants and ascertain the cause. The reasons for loss-to-follow-up were properly recorded.

Pulmonary function tests were performed according to the American Thoracic Society guidelines using the Vmax 22 (Sensor Medics, Yorba Linda, CA, USA) and PFDX (Medgraphics, St Paul, MN, USA)^25^. Post-bronchodilator FEV1 and FVC, indices of lung volume, as well as the diffusing capacity of carbon monoxide (DLCO), were measured at baseline and at each annual visit. All post-bronchodilator spirometry values were measured 15 min after the administration of 400 µg of salbutamol. Bronchodilator reversibility was identified when the FEV1 increased by 12% and at least 200 mL above the baseline value after salbutamol administration^11^. Static lung volumes, total lung capacity (TLC), and residual volume (RV) were measured using body plethysmography with a V6200 (CareFusion, San Diego, CA, USA), Vmax 22, or PFDX^26^.

Volumetric CT scans were taken at the time of enrollment, after 1 year, and then at intervals of 3 years. The scans were taken at full inspiration and expiration using 16-multidetector CT scanners (Somatom Sensation 16; Siemens Medical Systems, Bonn, Germany; GE Lightspeed Ultra; General Electric Healthcare, Milwaukee, WI, USA; Philips Brilliance 16; Philips Medical Systems, Best, Netherlands). Whole lung images were extracted automatically, and the attenuation coefficient of each pixel was calculated. The emphysema index was determined by calculation of the volume fraction of the lungs below −950 HU at full inspiration^27^.

### Statistical analyses

Characteristics were compared among groups using analysis of variance (for continuous variables) and chi-square tests, as appropriate. To additionally evaluate the difference in values between groups with further adjustments for possible confounding factors, analysis of covariance was used. When evaluating RV/TLC and emphysema index as continuous variables, the distributions of these data were assessed first. While RV/TLC showed normal distribution, emphysema index showed right-skewness. Accordingly, emphysema index was analyzed with log transformation. Cox-proportional hazard modeling was used to assess the relationship between groups in terms of the first event of acute exacerbation and mortality, with statistical adjustments for the following factors: age, sex, body mass index, pack-years smoked, smoking status, SGRQ score, exacerbation history at baseline, and use of long-acting β-agonists (ICS/LABA) or inhaled long-acting muscarinic antagonists (LAMA). In this way, we made more extensive adjustments for potential factors which may affect the clinical course of COPD compared to other major studies that assessed similar outcomes^28,29^. FEV1 was not included in the multivariate analyses because it was mutually correlated with both emphysema and RV/TLC. The proportional hazard assumptions of Cox regression models were assessed based on Schoenfeld residuals tests (Stata syntax “estat phtest”)^30^. All cox models did not show non-proportionality. Binomial negative regression analysis was used to assess the relationship between each group and annual exacerbation rates—defined in terms of incidence rate ratio (IRR)—with adjustments for the same covariates listed above. The 95% CIs were calculated, and p-values < 0.05 were considered statistically significant. All analyses were performed using IBM SPSS Statistics 20.0 (IBM Corp., Armonk, NY, US) and Stata, version 14.2 (StataCorp., College Station, TX, US).

## Results

### Patient characteristics

**Figure 1:**
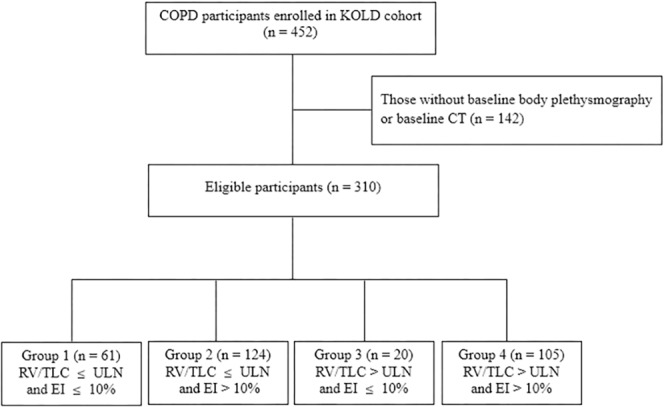
Flow diagram of the study population. Footnotes: RV/TLC, residual volume/total lung capacity ratio; ULN, upper limit of normal; EI, emphysema index.

A total of 310 subjects with COPD were eligible for analyses (Fig. 1). The mean (standard deviation) follow-up period was 61.1 (37.0) months. Table 1 shows a comparison of baseline demographic data and clinical characteristics among the groups. Compared to other groups, patients who had both resting hyperinflation and emphysema (Group 4) had the lowest BMI, FEV1 (in both the percentage of predicted value and z-score), and FEV1/FVC ratio, as well as the highest SGRQ score and mMRC grade.

**Table 1:** Baseline characteristics of patients with COPD classified in terms of the presence of resting hyperinflation and emphysema.

Characteristics	Group 1 (n = 61)	Group 2 (n = 124)	Group 3 (n = 20)	Group 4 (n = 105)	*P*-value^*^
Age	65.9 ± 7.9	66.1 ± 7.1	68.3 ± 7.6	66.8 ± 7.4	0.581
Men, n (%)	59 (96.7)	120 (96.8)	19 (95.0)	103 (98.1)	0.859
Body mass index, kg/m^2^	24.3 ± 3.1	22.9 ± 2.7	24.0 ± 3.4	21.6 ± 3.5	<0.001
Smoking status at baseline, n (%)					0.469
Current smokers	25 (41.0)	47 (37.9)	6 (30.0)	32 (30.5)	
Former smokers	36 (59.0)	77 (62.1)	14 (70.0)	73 (69.5)	
Pack-years of smoking	41.8 ± 21.7	47.8 ± 28.0	48.3 ± 35.4	45.3 ± 24.1	0.492
SGRQ score	32.0 ± 16.7	28.9 ± 16.5	34.2 ± 17.0	42.3 ± 17.6	<0.001
mMRC grade	1.5 ± 1.1	1.4 ± 1.0	1.9 ± 1.2	2.0 ± 1.0	<0.001
Chronic bronchitis, n (%)	21 (34.4)	29 (23.4)	6 (30.0)	29 (27.6)	0.460
**Lung function at baseline**					
FEV1, % predicted	68.4 ± 17.0	66.3 ± 15.3	57.8 ± 13.5	47.0 ± 16.7	<0.001
FEV1, z-score	−2.7 ± 1.4	−3.1 ± 1.2	−3.8 ± 1.1	−4.8 ± 1.2	<0.001
FVC, L	3.6 ± 0.8	3.9 ± 0.7	2.8 ± 0.6	3.1 ± 0.7	<0.001
FEV1/FVC	52.8 ± 9.7	47.2 ± 9.3	52.6 ± 9.1	40.3 ± 9.9	<0.001
DLCO, % predicted	87.9 ± 22.6	75.1 ± 21.5	94.0 ± 27.6	70.1 ± 24.1	<0.001
RV/TLC, %	36.9 ± 8.5	38.5 ± 9.2	58.8 ± 7.6	58.6 ± 7.6	<0.001
CT emphysema index, %	5.1 ± 3.0	25.9 ± 11.5	4.4 ± 3.0	32.5 ± 13.9	<0.001
Bronchodilator reversibility, n (%)	6 (9.8)	13 (10.5)	6 (30.0)	14 (13.3)	0.090
Eosinophil count, /uL	342.7 ± 554.7	257.1 ± 277.5	320.2 ± 266.8	248.6 ± 235.5	0.299
Exacerbation history, n (%)^†^	9 (14.8)	22 (17.7)	2 (10.0)	28 (26.7)	0.129
Exacerbation history, frequency‡	0.7 ± 2.3	0.6 ± 2.1	0.2 ± 0.6	0.6 ± 1.9	0.821

Group 1: RV/TLC ≤ upper limit of normal (ULN) and emphysema index ≤ 10%, Group 2: RV/TLC ≤ ULN and emphysema index >10%, group 3: RV/TLC > ULN and emphysema index ≤ 10%, group 4: RV/TLC > ULN and emphysema index >10%

SGRQ, St. George Respiratory Questionnaire; mMRC, modified Medical Round Council; FEV1, forced expiratory volume in one second; FVC, forced vital capacity; DLCO, diffusing capacity of carbon monoxide.

^*^P-values are for the overall comparison of the four groups and are based on analyses of variance and chi-square tests.

^†^History of ≥ 2 moderate, or ≥1 severe exacerbations in the past year.

^‡^The number of moderate or severe exacerbations in the past year.

Patients with resting hyperinflation had worse symptom grades than those without (mean SGRQ score: 29.9 ± 16.6 in Groups 1 and 2 vs. 41.0 ± 17.7 in Groups 3 and 4, p < 0.001; mean mMRC grade: 1.4 ± 1.0 in Groups 1 and 2 vs. 2.0 ± 1.0 in Groups 3 and 4, p < 0.001). This difference in symptom grades remained statistically significant after adjustment for following factors: age, sex, body mass index, pack-years smoked, smoking status, exacerbation history at baseline. Conversely, the relationship between emphysema and symptoms was relatively weak regardless of adjustments. Similarly, patients with resting hyperinflation had more severe airflow limitation, as measured by FEV1, regardless of whether they had emphysema.

### Respective associations of resting hyperinflation and emphysema with outcomes of COPD

Table 2 shows the respective associations of resting hyperinflation and emphysema with exacerbations over time; these were estimated by the hazard ratio (HR) for time to first exacerbation, and the incidence rate ratio (IRR) for exacerbation incidences. After adjustment for potential confounding factors, elevated RV/TLC as a continuous variable was significantly associated with (1) a higher risk of an earlier exacerbation event (HR = 1.02, 95% CI = 1.01–1.03) and (2) a higher incidence rate of exacerbation (IRR = 1.01, 95% CI = 1.002–1.03). As a categorical variable, patients with resting hyperinflation had a significantly higher risk of an earlier exacerbation event (HR = 1.66, 95% CI = 1.24–2.22) and a higher incidence rate of exacerbation (HR = 1.35, 95% CI = 1.01–1.81). The emphysema index showed trends that were similar to those of the RV/TLC. Elevated emphysema index (in log scale) as a continuous variable was significantly associated with a higher risk of an earlier exacerbation event (HR = 1.45, 95% CI = 1.01–2.09). Patients with emphysema had a significantly higher risk of an earlier exacerbation event (HR = 1.64, 95% CI = 1.15–2.35). The associations with mortality are shown in Table 3. Resting hyperinflation and emphysema were significantly associated with higher mortality risk, respectively.

**Table 2:** Unadjusted and adjusted associations of resting hyperinflation and emphysema with acute exacerbation in COPD.

Parameters	Unadjusted effect (95% CI)	Adjusted^*^ effect (95% CI)	*P*-value for adjusted effect
**RV/TLC**			
**As continuous variable, X + 1** ***vs*** **X %**			
HR^†^	1.03 (1.02–1.04)	1.02 (1.01–1.03)	0.001
IRR^‡^	1.03 (1.02–1.04)	1.01 (1.002–1.03)	0.024
**As categorical variable, >ULN (Resting hyperinflation)** ***vs*** ** ≤ ULN**			
HR^†^	1.93 (1.45–2.56)	1.66 (1.24–2.22)	0.001
IRR^‡^	1.81 (1.35–2.43)	1.35 (1.01–1.81)	0.046
**Emphysema index**			
**As continuous variable, X + 1** ***vs*** **X (in log scale)**			
HR^†^	1.67 (1.19–2.36)	1.45 (1.01–2.09)	0.044
IRR^‡^	1.67 (1.24–2.25)	1.10 (0.79–1.51)	0.579
**As categorical variable, >10% (Emphysema)** ***vs*** ** ≤ 10%**			
HR^†^	1.59 (1.13–2.23)	1.64 (1.15–2.35)	0.007
IRR^‡^	1.72 (1.23–2.42)	1.26 (0.91–1.75)	0.167

^*^Adjusted by age, sex, body mass index, pack-years smoked, smoking status, SGRQ score (25 vs <25), exacerbation history (≥2 total or ≥1 severe exacerbations vs <2 total and 0 severe exacerbation) in the past year at baseline, and use of ICS/LABA or LAMA.

^†^Hazard ratio for time to 1st acute exacerbation.

^‡^Incidence rate ratio for exacerbation frequency (total counts/person-years).

**Table 3:** Unadjusted and adjusted associations of resting hyperinflation and emphysema with mortality in COPD.

Parameters	HR	95% CI	*P*-value
**RV/TLC**			
**As continuous variable, X + 1** ***vs*** **X %**			
Unadjusted	1.06	1.03–1.08	<0.001
Adjusted^*^	1.02	0.99–1.04	0.221
**As categorical variable, >ULN (Resting hyperinflation)** ***vs*** **ULN**			
Unadjusted	4.28	2.12–8.64	<0.001
Adjusted^*^	2.45	1.16–5.17	0.019
**Emphysema index**			
**As continuous variable, X + 1** ***vs*** **X (in log scale)**			
Unadjusted	6.37	2.23–18.21	0.001
Adjusted^*^	2.96	1.07–8.17	0.036
**As categorical variable, >10% (Emphysema)** ***vs*** **≤10%**			
Unadjusted	3.66	1.31–10.19	0.013
Adjusted^*^	3.13	1.06–9.27	0.039

^*^Adjusted by age, sex, body mass index, pack-years smoked, smoking status, SGRQ score (25 *vs* <25), exacerbation history (≥ 2 total or ≥1 severe exacerbations *vs* <2 total and 0 severe exacerbation) in the past year at baseline, and use of ICS/LABA or LAMA.

### Combined associations of resting hyperinflation and emphysema with outcomes of COPD

**Figure 2:**
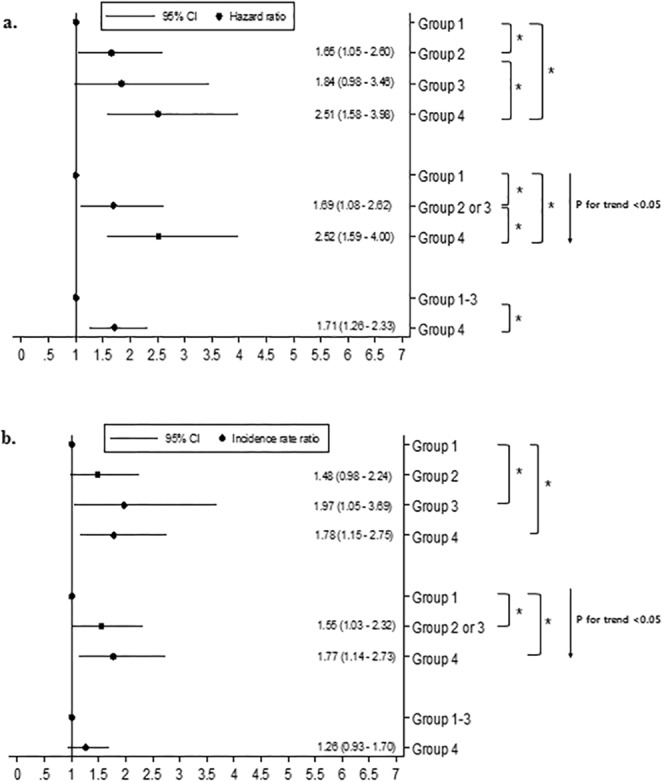
The combined associations of resting hyperinflation and emphysema with acute exacerbation. (**a**) Time to first acute exacerbation. Footnotes: Group 1: RV/TLC ≤ upper limit of normal (ULN) and emphysema index ≤  10%, Group 2: RV/TLC ≤ ULN and emphysema index >10%, group 3: RV/TLC > ULN and emphysema index ≤ 10%, group 4: RV/TLC > ULN and emphysema index >10%. All statistical analyses were adjusted by age, sex, body mass index, pack-years smoked, smoking status, SGRQ score (≥25 vs <25), exacerbation history (≥2 total or ≥1 severe exacerbations vs <2 total and 0 severe exacerbation) in the past year at baseline, and use of ICS/LABA or LAMA. *P-value < 0.05. (**b**) Incidence rate of acute exacerbation. Footnotes: Group 1: RV/TLC ≤ upper limit of normal (ULN) and emphysema index ≤ 10%, Group 2: RV/TLC ≤ ULN and emphysema index >10%, group 3: RV/TLC > ULN and emphysema index ≤ 10%, group 4: RV/TLC > ULN and emphysema index >10%. All statistical analyses were adjusted by age, sex, body mass index, pack-years smoked, smoking status, SGRQ score (≥25 vs <25), exacerbation history (≥2 total or ≥1 severe exacerbations vs <2 total and 0 severe exacerbation) in the past year at baseline, and use of ICS/LABA or LAMA. ^*^P-value < 0.05.

**Figure 3:**
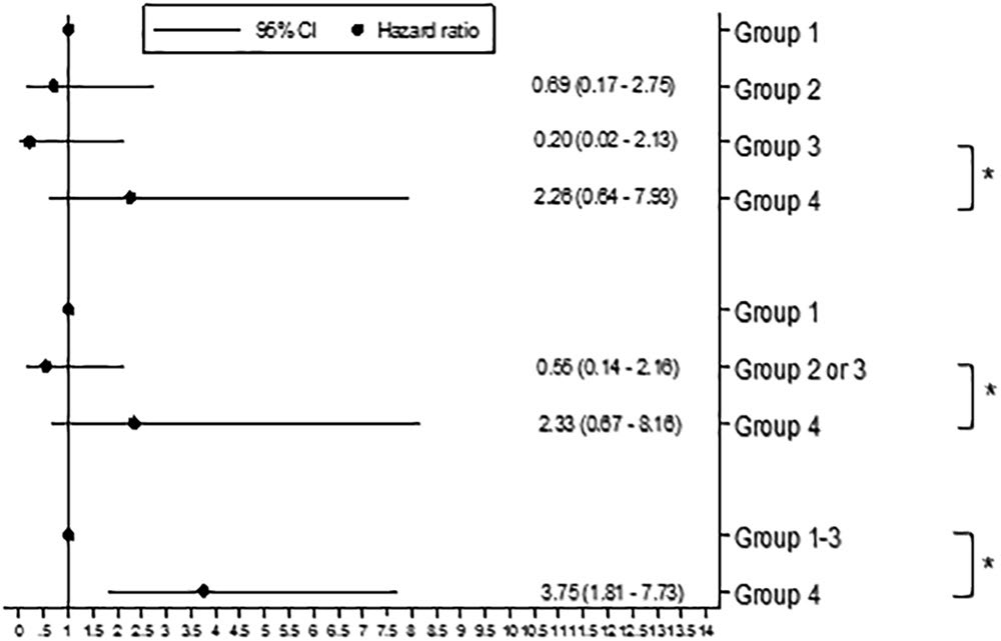
The combined associations of resting hyperinflation and emphysema with mortality in patients with COPD. Footnotes: Group 1: RV/TLC ≤ upper limit of normal (ULN) and emphysema index ≤ 10%, Group 2: RV/TLC ≤ ULN and emphysema index >10%, group 3: RV/TLC > ULN and emphysema index ≤ 10%, group 4: RV/TLC > ULN and emphysema index >10%. All statistical analyses were adjusted by age, sex, body mass index, pack-years smoked, smoking status, SGRQ score (≥25 vs <25), exacerbation history (≥2 total or ≥1 severe exacerbations vs <2 total and 0 severe exacerbation) in the past year at baseline, and use of ICS/LABA or LAMA. ^*^P-value < 0.05.

Figure 2a (time to first exacerbation) and 2b (exacerbation rates) show the comparison of exacerbation risk among the groups. Patients who had both resting hyperinflation and emphysema (Group 4) had a significantly higher risk of exacerbations than those with neither (Group 1; HR = 2.51, 95% CI = 1.58–3.98; IRR = 1.78, 95% CI = 1.15–2.75). In addition, patients with both resting hyperinflation and emphysema (Group 4) showed a significantly higher risk of exacerbations than all other groups (Groups 1–3; HR = 1.71, 95% CI = 1.26–2.33). The trend of increasing risk from group 1 (no feature) to group 2 or 3 (1 feature) to group 4 (2 features) was statistically significant for both time to first exacerbation (HR = 1.56, 95% CI = 1.26–1.94) and exacerbation rate (IRR = 1.29, 95% CI = 1.04–1.60). Figure 3 shows the comparison of mortality risk among the groups. Patients who had both resting hyperinflation and emphysema (Group 4) had a significantly higher risk of mortality than other groups (Groups 1–3; HR = 3.75, 95% CI = 1.81–7.73).

## Discussion

The present study explored the independent and combined associations of resting hyperinflation and emphysema with the clinical characteristics and outcomes of COPD. The results revealed that clinical symptoms, as measured by SGRQ and mMRC scores, are more closely related to resting hyperinflation than to emphysema. Both resting hyperinflation and emphysema were independent predictors of exacerbation risk and mortality. In addition, patients with both resting hyperinflation and emphysema had a significantly higher risk of exacerbation and mortality than those with only one or neither of the conditions. To our knowledge, this was the first study to identify the combined association of resting hyperinflation and emphysema with the clinical outcomes of COPD. Our results add novel information to the field and indicate that careful inspection of both features in combination could provide additional information that is useful for predicting the disease course of COPD in clinical practice.

It is widely accepted that small airway disease and emphysema are the two main components of COPD pathology, and that various severities and combinations of these components constitute the spectrum of disease phenotypes. It follows that proper classification of the underlying COPD phenotypes would improve prognostic predictions and enable individualized treatment^6^. Specifically, resting hyperinflation is caused by increased compliance and loss of elastic recoil in the lung; these effects are in turn a result of injury to the small airways and parenchymal destruction. Thus, resting hyperinflation is not necessarily equivalent to emphysema^7^. To date, the degree of resting hyperinflation is most commonly measured using indices from body plethysmography, elevated RV/TLC is a typical indicator^22^. The results of the present study concur with those of previous studies that have suggested a close relationship between resting hyperinflation and the clinical symptoms of COPD^9,31^. In addition, our results provide data indicating that an elevated RV/TLC is an independent risk factor for exacerbations and mortality even with extensive adjustments for potential factors that may also influence the clinical course. This supports and extends the knowledge regarding resting hyperinflation as a prognostic factor in COPD^13,14^.

The present study also indicated that resting hyperinflation is more important than emphysema for understanding the status and course of COPD. In terms of the relationships between emphysema and exacerbations, our study was consistent with previous studies, which have suggested that the presence of emphysema is a risk factor for exacerbations^19,32^. However, it is notable that, according to our results, the relationship between emphysema and clinical symptoms and its value in predicting exacerbations were relatively weak compared with those of resting hyperinflation. Specifically, our results showed that patients with resting hyperinflation had more severe airflow limitation, regardless of whether they had emphysema. The association with resting hyperinflation independent of emphysema could be explained by the contribution of small airway disease. This supports the concept that small airway injury precedes emphysema with increasing severity of COPD^33,34^.

In addition to small airway injury and parenchymal destruction due to COPD, resting hyperinflation can also result from normal aging^35^. Physiological studies have shown that, with advancing age, progressive enlargement of the alveolar ducts, as well as changes in the collagen and elastin content of the airways, lead to loss of elastic recoil and hyperinflation. The same changes are present in patients with COPD^36,37^. Indeed, a recent study by Martinez *et al*. showed that, in normal subjects without COPD, aging resulted in increased hyperinflation associated with small airway dysfunction. Conversely, emphysema was not associated with aging in non-smokers^15^. Aging can be a significant confounding factor during the evaluation of COPD status based the on degree of hyperinflation alone. Therefore, we can assume that the combined inspection of hyperinflation with emphysema, which is a distinct pathological process of COPD, provides additional information regarding the prediction of disease status and prognosis. Similarly, a recent study by Galban *et al*. reported that simultaneous evaluation of emphysema and hyperinflation, as determined by inspiratory and expiratory CT imaging, respectively, could more objectively characterize and classify the clinical phenotypes of COPD^34^. However, expiratory CT images are not yet widely obtained from all patients in real clinical practice, and they increase the amount of radiation exposure^38^. Therefore, RV/TLC from body plethysmography may be a reliable alternative for measuring resting hyperinflation. Our results support the hypothesis that evaluation of resting hyperinflation and emphysema in combination confers additional information for predicting exacerbations and mortality in the clinical course of COPD. They also indicate that, in routine practice, both RV/TLC and the emphysema index should be closely monitored and interpreted in combination.

The main strength of our study is the use of a well-designed, prospective cohort that included subjects with COPD at various stages of severity. The cohort was purely observational; thus, the events recorded were likely to represent disease-related changes in patients who were properly managed. Moreover, because longitudinal and accurate data on exacerbation and mortality were collected over a long observational period, the prognostic value of the analyzed indices could be evaluated adequately. In addition, our analyses, we performed extensive adjustments for factors that may also influence the clinical course of COPD, suggesting that our results have high validity.

Our study had several limitations. First, although the prediction equations used to evaluate the upper limit of normality of RV/TLC and the cutoff value for emphysema index used in the present study are generally accepted^10,17,25^, the accumulated evidence on the exact cutoff value for these indices remains weak, especially when regarding possible variation between races. Second, although the protocol of our study included evaluation of CT indices at expiration, no factors from expiratory CT exams were significantly associated with COPD outcomes (data not shown). Third, in the KOLD cohort study, the reproducibility or variability of CT density obtained from different clinics was not measured. However, all CT scans were performed with a prescribed protocol, and images were sent to a central lab to be checked for consistency^23^. Fourth, we did not apply random effects from institutions in the statistical analyses. Although major observational studies generally present results analyzed regardless of random effects from centers^39,40^, possible imprecisions may exist. Fifth, because the study was mainly focused on the association between baseline clinical values and subsequent outcomes, the longitudinal aspects of variables were not significantly considered. Moreover, possible residual confounding such as influence of age on mortality could also be a limitation of our study.

In conclusion, combined evaluation of resting hyperinflation and emphysema using body plethysmography and CT confers additional information and better prediction of the clinical course of COPD. It may also contribute to a more adequate phenotypical classification of COPD.
